# Heat stress induces proteomic changes in the liver and mammary tissue of dairy cows independent of feed intake: An iTRAQ study

**DOI:** 10.1371/journal.pone.0209182

**Published:** 2019-01-09

**Authors:** Lu Ma, Yongxin Yang, Xiaowei Zhao, Fang Wang, Shengtao Gao, Dengpan Bu

**Affiliations:** 1 State Key Laboratory of Animal Nutrition, Institute of Animal Science, Chinese Academy of Agricultural Sciences, Beijing, China; 2 Institute of Animal Science and Veterinary Medicine, Anhui Academy of Agricultural Sciences, Hefei, China; 3 CAAS-ICRAF Joint Lab on Agroforestry and Sustainable Animal Husbandry, World Agroforestry Centre, East and Central Asia, Beijing, China; 4 Hunan Co-Innovation Center of Animal Production Safety, CICAPS, Changsha, Hunan, China; University of Illinois, UNITED STATES

## Abstract

Heat stress decreases milk yield and deleteriously alters milk composition. Reduced feed intake partially explains some of the consequences of heat stress, but metabolic changes in the mammary tissue and liver associated with milk synthesis have not been thoroughly evaluated. In the current study, changes of protein abundance in the mammary tissue and liver between heat-stressed cows with ad libitum intake and pair-fed thermal neutral cows were investigated using the iTRAQ proteomic approach. Most of the differentially expressed proteins from mammary tissue and liver between heat-stressed and pair-fed cows were involved in Gene Ontology category of protein metabolic process. Pathway analysis indicated that differentially expressed proteins in the mammary tissue were related to pyruvate, glyoxylate and dicarboxylate metabolism pathways, while those in the liver participated in oxidative phosphorylation and antigen processing and presentation pathways. Several heat shock proteins directly interact with each other and were considered as central “hubs” in the protein interaction network. These findings provide new insights to understand the turnover of protein biosynthesis pathways within hepatic and mammary tissue that likely contribute to changes in milk composition from heat-stressed cows.

## Introduction

Heat stress detrimentally affects a variety of economically important production traits and thus results in enormous economic losses on the global dairy industry. During heat stress, dairy cows experience a series of physiological and behavioral responses that are presumably survival strategies employed to ensure successful adaptation to a heat load. In addition to overall milk yield, milk fat and protein content are annually decreased during the hot summer months [1–4], and we have also reported that heat stress has direct effects on milk yield and milk protein content [5].

Milk synthesis and secretion are considered system processes incredibly sensitive to both physiological and environmental factors. Decreased milk yield and changes in milk component content were traditionally thought to result from decreased nutrient intake [3], but recent experiments are utilizing a pair-feeding model which demonstrates that inadequate feed intake only accounts for about 50% of the decrease in milk synthesis among heat stressed cows [6–8]. Thus, changes in postabsorptive metabolism and nutrient partitioning may contribute to discordant changes in mammary protein synthesizing capacity in heat-stressed cows. In addition, recent reports indicate that heat stress decreases the expression of major milk protein genes, increases the expression of several chaperone genes, induces apoptosis and interferes with cytoskeletal and cell transport function in mammary epithelial cells among heat stressed cows [9–11]. Milk production is reliant on the normal structure and function of mammary epithelial cells that synthesize and secrete milk [12–13] and these heatstress studies indicate that changes in overall and milk component synthesis in heat-stressed cows are partially caused by changes in specific regulation of milk synthesis, rather than just a general reduction in milk activity. From a milk protein perspective, the proportion of whey is increased and casein fractions are decreased in heat-stressed compared to pair-fed cows [6].

The liver and mammary tissue have complementary and coordinated metabolic roles during lactation [14]. For example, hepatic derived glucose (rates of which are sensitive to lactational needs) is taken up by the mammary tissue and used for multiple milk synthesizing processes; most notably lactose production, the primary osmorugulator of milk volume [15–16]. Further, amino acids, triglycerides and ketones are exported from the liver and are key precursors for milk synthesis [6,17–19]. Moreover, circulating heat shock proteins (HSP) and AMP-activated protein kinase were increased in heat-stressed cows [20–21]. As well, several variables associated with systemic energy and protein metabolism are affected by heat stress [8,22–23]. However, few experiments have focused on proteomic changes in the liver or mammary tissue, which could partially explain the negative effects of heat stress on productivity.

Thus, we hypothesized that the endogenous protein profiles in the mammary tissue and liver of lactating dairy cows are altered by heat stress independent of reduced feed intake. Study objectives were to investigate heat-stressed induced changes in mammary tissue and liver proteins by iTRAQ approach. Results of this study may provide novel information to explore the molecular mechanism of protein biosynthesis in the liver and mammary tissue contributed to milk components of dairy cows independent of reduced feed intake under the heat stress.

## Materials and methods

### Animals and experimental treatments

All animal care and procedures were approved by the Animal Care Advisory Committee of the Chinese Academy of Agricultural Sciences. Four multiparous Chinese Holstein cows (101±10 DIM, 574±36 kg BW, 38±2 kg milk/d, 2nd parity) were individually housed in environmental chambers at Changping Research Station (Beijing, China), fed a TMR twice daily at 0500 and 1700 h, and orts were recorded daily before the morning feeding. Cows were milked twice daily at 0500 and 1700 h and milk yields were recorded at each milking. The TMR containing alfalfa hay, extruded-soybean, whole corn silage, steam-flaked corn, bean pulp, rapeseed meal and feeding corn meal was formulated to meet the predicted requirements (NRC, 2001) of energy, protein, minerals, and vitamins (S1 Table).

Cows were acclimated for 9 days with ad libitum access to feed and water in thermal neutral conditions of 20°C, 55% humidity, and 12 h light and dark cycles (temperature-humidity index (THI) = 65). Cows were then randomly assigned into a two-treatment crossover design study. The study was comprised of 2 experimental periods with each of 9 days and a 30 days thermal neutral washout period. During the period of heat stress induction, the two heat-stressed cows were individually housed in a climatic chamber and were fed ad libitum with the temperatures ranging from 32 to 36°C, with 55% humidity, and 12 h light and dark cycles (temperature-humidity index (THI) = 65). Between 0600 and 1800 h, the temperature was kept at 36°C, and between 1800 and 0600 h, the temperature was kept at 32°C. The other two pair-fed cows were also individually housed in a climatic chamber under thermal neutral conditions (as above) but were fed to match the feed intake of the heat-stressed cows. Dry matter intake (DMI) was measured for each cow once daily [5]. And all data of THI and DMI were statistically analyzed utilizing SAS version 9.3 (SAS Institute Inc., Cary, NC) with mixed model.

### Sample collection and protein preparation

Milk samples from individual cows were collected in the morning and evening milking throughout the experimental periods. Daily milk samples from each cow were pooled. Bronopol tablet (D&F Control System, San Ramon, CA) was added and stored at 4°C until analysis using a Milk Oscan Minor machine (MilkoScan Type 78110, Foss Electric, Hillerød, Denmark). Mammary and liver tissue needle biopsies were collected from individual cows on the last day of each environmental period as described in previous studies [24–25]. Samples were washed with precooled PBS at 4°C, cut into 4–5 slices (approximately 100 mg each slice) and stored in liquid nitrogen. Unexpectedly, a liver sample could not be harvested from one of the cows due to technical issues and therefore liver and mammary tissues from only 3 cows were used for the proteomic analysis for each group (pair-fed and heat-stressed).

About 100 mg of mammary or liver tissue samples were placed in a tube and mixed with 1mL lysis buffer (4% SDS and 0.1 M DTT in 0.1 M TrisHCl, pH 7.6), and then mixed with quartz sand at room temperature [26]. Samples were homogenized with 65.0 Hz in a MP FastPrep for 3 min. After samples were incubated for 20 min at 95°C, homogenates were sonicated for 2 min at 200W and then centrifuged at 16 000 × g for 30 min to obtain the supernatant. Protein concentrations were determined by a bicinchoninic acid assay (Pierce, Rockford, IL, USA). Samples were then stored at -80°C [27].

### Protein digestion

One hundred micrograms of the protein sample was mixed with dithiothreitol solution at a final concentration of 100 mM and then incubated for 5 min at 95°C. Two hundred mL of 8 M urea and 150 mM Tris-HCl, pH 8.0 were added after the samples had cooled to room temperature. The mixtures were transferred to an ultrafiltration filter (10-kDa cutoff, Sartorius, Germany) and centrifuged at 14 000 × g for 15 min. Subsequently, 100 μL of iodoacetamide solution (50 mM iodoacetamide in 8 M urea and 150 mM Tris-HCl, pH 8.0) was added and incubated for 30 min at room temperature in the dark. Then 100 μL dissolution buffer (Applied Biosystems, Foster City, CA, USA) was added, mixed and centrifuged at 14 000 × g for 10 min and this step was repeated twice. Finally, 20 μL trypsin solution (2 μg trypsin in 20 μL dissolution buffer) was added, mixed and incubated for 16–18 h at 37°C. The digested peptides were collected into a new tube by centrifugation at 14 000 × g for 10 min.

### iTRAQ labeling

Digested peptides from mammary and liver were labeled with iTRAQ reagents based on the manufacturer instructions (Applied Biosystems, USA). Mammary and liver samples from control cows were labeled with reagent 115, 117 and 119, and heat-stressed cows labeled with reagent 116, 118 and 121. The mixtures were incubated for 1 h at room temperature to perform labeling reaction.

### Reverse phase high performance liquid chromatography separation

The labeled peptides were separated by an Agilent 1100 HPLC (Agilent Technologies, Paulo Alto, CA USA). The chromatography column (2.0 × 50 mm, 5 μm, XBridge BEH 300 C18, Waters, Ireland) was equilibrated by solution A consisted of ammonia water (pH 10.0). The peptides were separated with solution B consisted of 100% acetonitrile (pH 10.0) as follow: 0 to 5% (v/v) for 2 min, 5 to 35% (v/v) for 58 min, 35 to 50% (v/v) for 10 min, 50 to 90% (v/v) for 4 min, and 90% (v/v) for 2 min at a flow rate of 500 μL/min. One fraction was collected every 2 min. A total of 40 sub-fractions were collected and pooled into 20 fractions. Samples were desalted on the C18 solid phase extraction column.

### Mass spectrometry analysis

Peptides fractions were further separated and analyzed by a Thermo Fisher EASY-nLC 1000 system coupled with a Q-Exactive (Thermo Fisher Scientific, San Jose, CA, USA). Capillary column was equilibrated with 95% (v/v) solution C consisted of 0.1% (v/v) formic acid in MilliQ water, and then samples were loaded onto the trap column (2 cm × 100 μm, 5 μm) by an autosampler. The labeled peptides were separated on the reverse-phase column (100 mm × 75 μm, 3 μm) with solution D consisted of 0.1% (v/v) formic acid and 90% (v/v) acetonitrile in MilliQ water at a flow rate of 300 nL/min. The segmented gradient was run from 2% to 5% (v/v) for 2 min, from 5% to 35% (v/v) for 42 min, from 35% to 90% (v/v) for 3 min and then 90% (v/v) solution D for 3 min.

Q-Exactive (Thermo Fisher Scientific) was used to perform peptide analysis in positive ion mode with a selected precursor ions range of 300–1800 m/z. For the survey scan, resolving power was set to 70 000 at m/z 200. The top 12 most abundant precursor ions with charge ≥ 2 were selected by each survey scan cycle and fragmented by higher-energy collisional dissociation with normalized collision energy of 30 *eV*. For the MS/MS scans, resolving power was set to 17,500 at m/z 200. Capillary column temperature was set at 270°C, automatic gain control target value at 3E6 and dynamic exclusion at 30 s. The underfill ratio was determined at 0.1% [28].

### Protein identification and quantification

Raw files were processed in MaxQuant 1.5.2.8 and then used to search an in-house uniprot database of bovine with 32246 entries (06–2015). The searching parameters were monoisotopic mass, trypsin as the enzyme and allowing up to two missed cleavages. Fragment and peptide mass tolerance were set at 0.1 Da and 20 ppm, respectively. Oxidation of methionine and acetylation of protein N-term were specified as variable modifications. Carbamidomethylation of cysteine, iTRAQ-labeled N-terminus and iTRAQ-labeled lysine were defined as fixed modifications. Protein and peptide identifications were based on 99% confidence as determined by a false discovery rate of no more than 1%.

Relative quantification of the identified proteins was performed by MaxQuant software. Relative peak intensities of released iTRAQ reporter ions were used to calculate the relative ratios of identified peptides. The weighted ratios of uniquely identified peptides from the specific protein were used to calculate the relative quantification of the individual proteins. Final ratios of the individual proteins were normalized based on the median average quantification ratio for all labeled samples. Protein identification was accepted at least two unique peptides that were used to the further analysis. Quantified proteins were analyzed by independent T-test, and their fold changes were calculated. Differentially expressed proteins were those with *P* < 0.05 and fold change > 1.2 between groups [29].

### Western blot analysis

Each sample was mixed with 5× sample loading buffer and boiled at 95°C for 8 min, and was separated on a 10% SDS-PAGE gel, then transferred onto nitrocellulose membranes using 200 mA of constant current. Membranes were then incubated with primary antibodies specific (HSP 90α/β, Santa Cruz Biotechnology, SC-59578, Achlya Ambisexualis Origin, Monoclonal, USA) for target proteins for 2 h at room temperature. Subsequently, membranes were washed three times with TBST. Then membranes were incubated with secondary antibody (Anti-Mouse produced in rabbit, SAB3701212, Sigma) for 2 h at room temperature followed by the same wash step. Membranes were visualized with ECL Western Blotting Substrate. β-Actin was used as an internal reference protein to normalize protein expression. The images were scanned with a GS-800 Calibrated Densitometer (Bio-Rad, Hercules, CA, USA), and the lanes were analyzed with Image J software.

### Data analysis

Functional analysis of the identified proteins from mammary and liver was performed by DAVID Bioinformatics Resources (http://david.abcc.ncifcrf.gov/home.jsp). Differential expressed proteins were imported into the online Search Tool for the Retrieval of Interacting Genes/Proteins (STRING) software (http://string-db.org) to predict protein interactions [30]. Quantified proteins were processed using Cluster 3.0 software to investigate the hierarchical clustering of the identified proteins. Java TreeView was used for data visualization.

## Results

### DMI and milk composition

Although DMI was similar between pair-fed and heat-stressed cows (by experimental design), milk yield was significantly decreased in heat-stressed cows comparative with pair-fed cows. Milk protein content was different between two groups (Table 1), while milk fat content was not different between two groups.

**Table 1:** THI and effects of pair-fed or heat stress on feed intake, milk yield and milk compositions.

Items	Treatment		SEM	*P* value			
	PF^4^	HS^5^		Trt^1^	Day	T×D^2^	Per×Trt^3^
THI	64.6	82.4	0.34	< .0001	0.6662	0.5899	0.9623
DMI, Kg	10.1	10.2	0.94	0.8922	< .0001	1.0000	0.6542
Milk yield, Kg/d	25.9	21.5	1.22	0.0372	< .0001	0.5158	0.0774
Protein, %	2.68	2.57	0.021	0.0149	0.0003	0.4408	0.4278
Fat, %	4.28	4.13	0.291	0.6557	0.0223	0.9315	0.1889
Lactose, %	4.79	4.66	0.102	0.3055	0.0016	0.5469	0.1135
Milk solid not fat, %	8.03	7.77	0.098	0.0765	0.0002	0.0035	0.8140

^1^Treatment.

^2^Treatment by day interaction.

^3^Period by treatment interaction.

^4^Pair-fed thermoneutral.

^5^Heat stress.

### Statistical analysis of identified proteins

A total of 2,789 proteins in mammary tissue and 2,781 proteins in liver were identified through peptide identification. These identified proteins from mammary tissue and liver were also quantified by the iTRAQ proteomic approach listed in S2 and S3 Tables. 80 proteins in mammary tissue and 200 proteins in liver were differed in abundance between heat-stressed and pair-fed thermal neutral control cows, respectively. In mammary tissue, several proteins were different including chitinase-3-like protein 1 and lysosomal protective protein that were significantly increased, while cytoplasmic malate dehydrogenase and signal recognition particle 19 kDa protein were decreased in heat-stressed cows compared with pair-fed control cows. In liver, several proteins belonging to the heat shock protein family were increased, while several subunits of NADH dehydrogenase complex were decreased in heat-stressed cows compared with pair-fed control cows.

Western blot of HSP 90α/β in liver and mammary gland showed differences between treatment groups (S1 Fig), which were consistent with proteomic results.

### Cluster analysis

**Fig 1:**
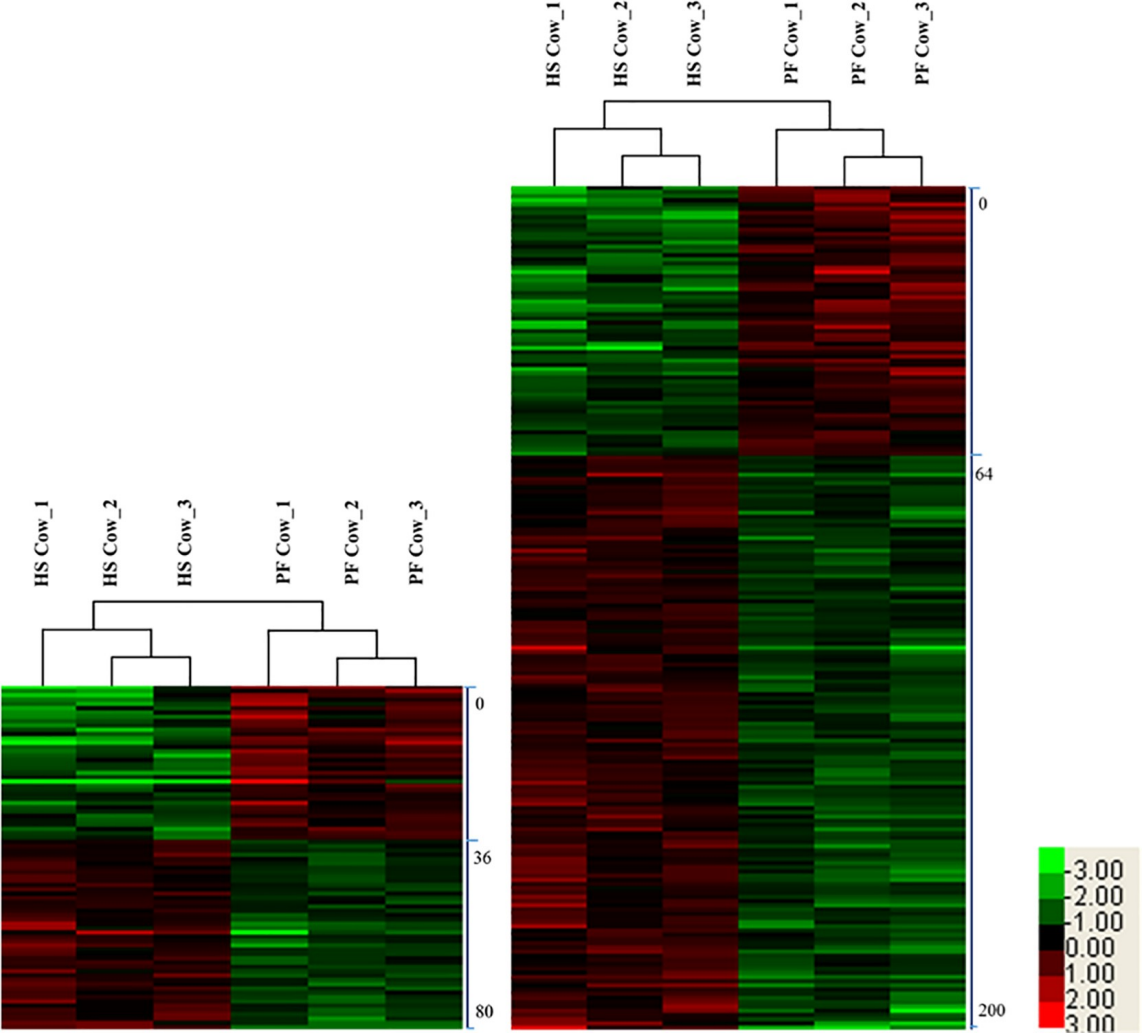
Hierarchical clustering of differentially expressed proteins in mammary gland (A) and liver (B) between heat-stressed and pair-fed cows. The bar color represents a logarithmic scale from -3.0–3.0. HS mean heat stress; PF mean pair-fed.

To gain insights into biological differences between heat-stressed and pair-fed cows, proteins which were quantified differentially from mammary tissue and liver were subjected to hierarchical clustering by Cluster 3.0 software (bonsai.hgc.jp/~mdehoon/software/cluster/software.htm). Hierarchical clustering analysis revealed that protein profiles could be used to accurately classify mammary tissue and liver samples from heat-stressed and pair-fed cows (Fig 1). For mammary tissue, cluster 1 including 36 proteins was significantly decreased and cluster 2 including 44 proteins was significantly increased in heat-stressed compared to control cows. For liver tissue, cluster 1 including 64 proteins was significantly decreased and cluster 2 including 136 proteins was significantly increased in heat-tressed compared to control cows.

### Functional and pathway analysis of the differentially expressed proteins

**Fig 2:**
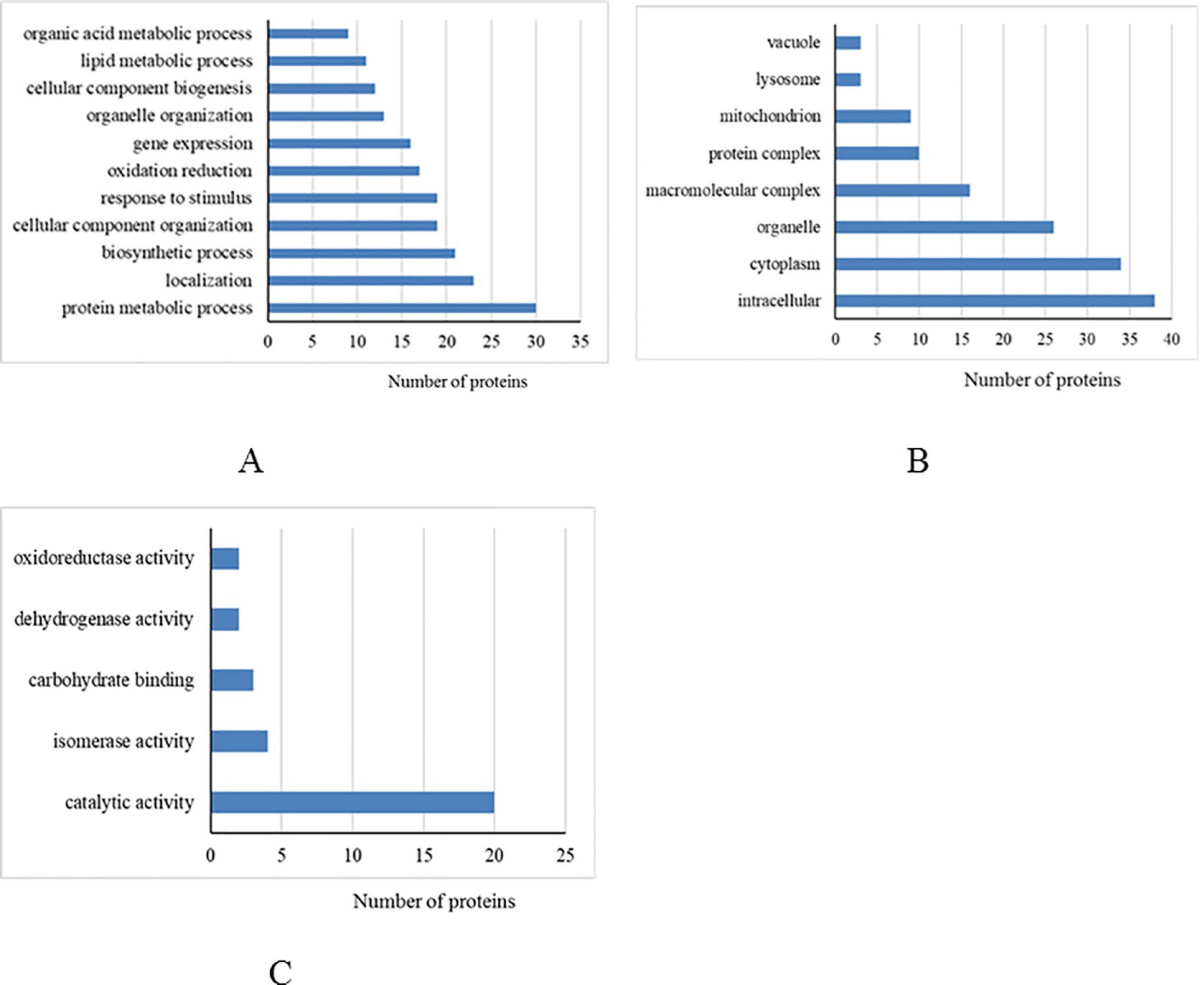
Differentially expressed proteins from mammary gland between heat-stressed and pair-fed control cows grouped into biological process (A), cellular component (B) and molecular function (C) based on David bioinformatics resources.

**Fig 3:**
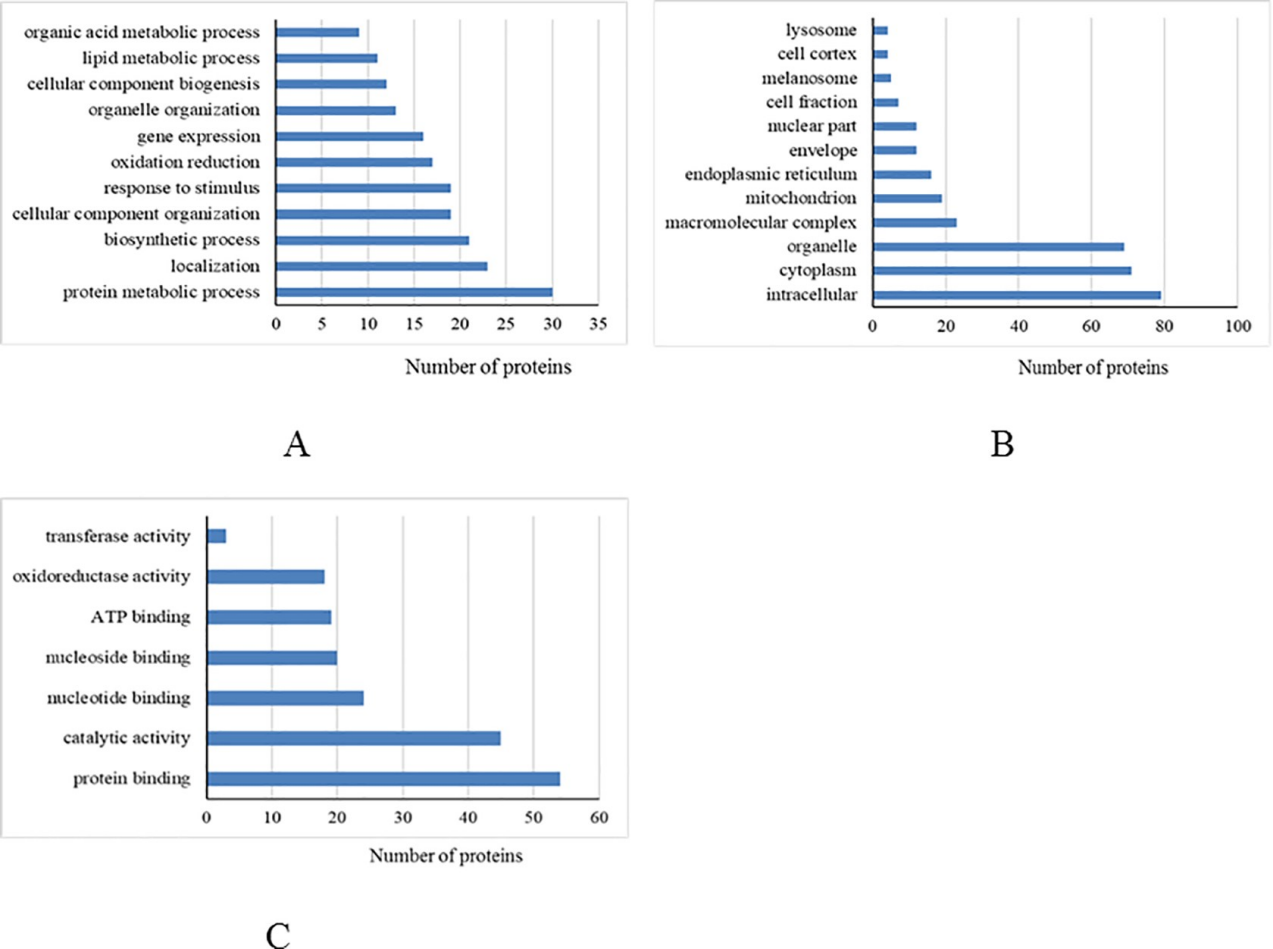
Differentially expressed proteins from liver between heat-stressed and pair-fed control cows grouped into biological process (A), cellular component (B) and molecular function (C) based on David bioinformatics resources.

Based on the annotated functions, differentially expressed proteins from the mammary tissue and liver were categorized into biological processes, cellular components, and molecular functions (Figs 2 and 3, respectively). We found that most of the differentially expressed proteins from the mammary tissue and liver between heat-stressed and pair-fed cows were located in the intracellular, cytoplasm and organelles that were involved in protein metabolic processes. For molecular function, most of the differentially expressed proteins in mammary tissue were associated with catalytic activity, while most differentially expressed proteins in liver were associated with protein binding.

Further, we found that several signaling pathways were altered by heat stress. Pyruvate metabolism, and glyoxylate and dicarboxylate metabolism pathways were significantly different in the mammary tissue, while antigen processing and presentation, and oxidative phosphorylation pathways were significantly different in the liver (Table 2).

**Table 2:** Pathway analysis of differential expressed proteins from mammary gland and liver between heat-stressed and pair-fed cows.

Tissues	Pathway Name	Count	Hits	*P* Value	Fold Enrichment
Mammary gland	Pyruvate metabolism	3	37	0.011	17.41
	Glyoxylate and dicarboxylate metabolism	2	11	0.048	39.05
Liver	Oxidative phosphorylation	7	130	0.004	4.51
	Antigen processing and presentation	4	67	0.043	5.00

### Protein interaction analysis of the differentially identified proteins

**Fig 4:**
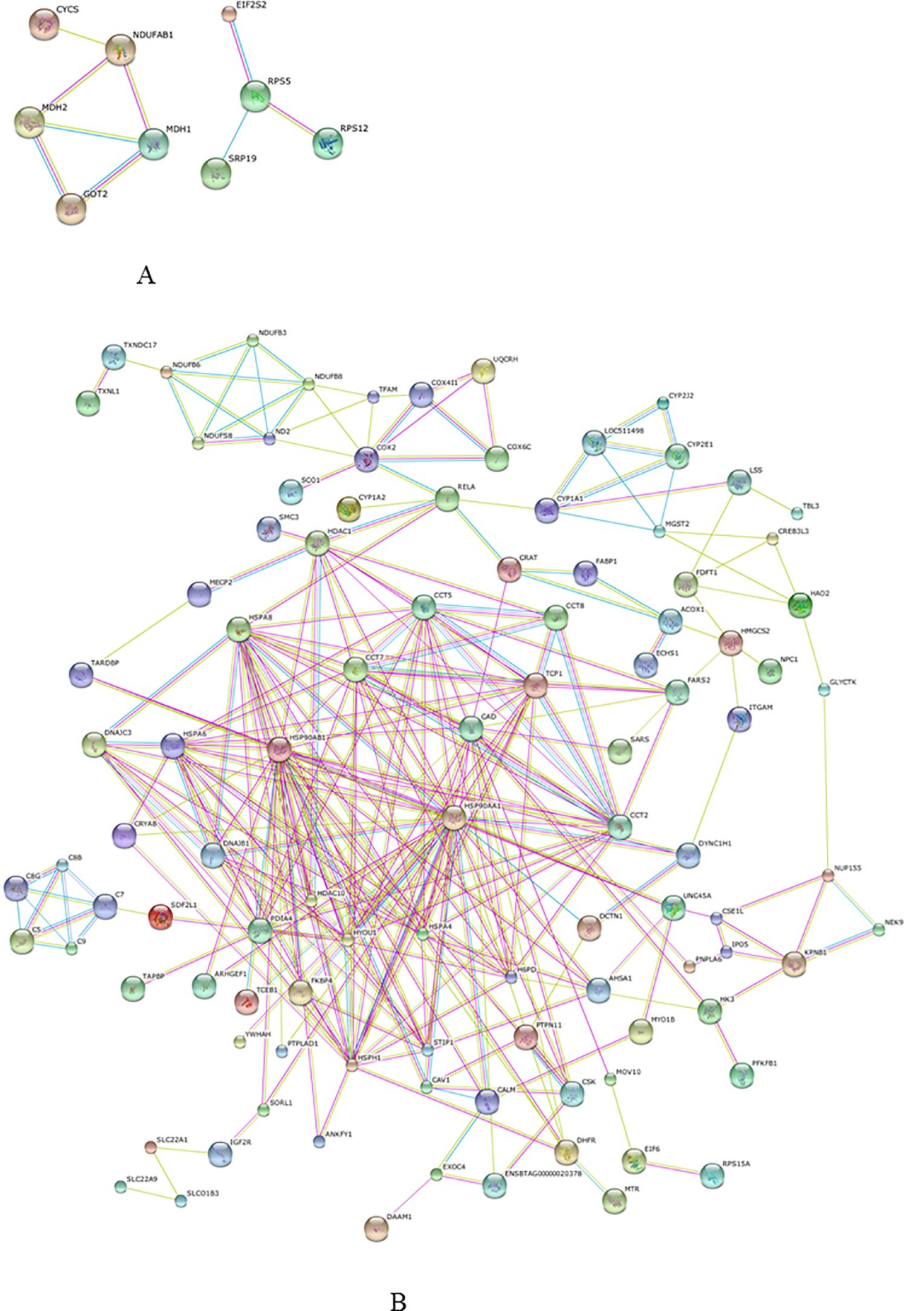
Protein interaction network of the differential proteins from mammary gland (A) and liver (B) predicted with STRING software. Each node presents a protein; line colors present the types of evidence: pink lines from experimental study, the blue lines from databases, and the yellow lines from abstracts of articles published in PubMed.

Protein-protein interactions of the differentially expressed proteins from the mammary tissue and liver were predicted by STRING software. In the mammary tissue, it was predicted that acyl carrier protein interacted with cytochrome c, cytoplasmic malate dehydrogenase (MDH1) and mitochondrial malate dehydrogenase (MDH2). In the liver, several HSPs including heat shock cognate 71 kDa protein, HSP 90-beta and HSP 90-alpha were directly interacted with each other and served as central “hubs” in the protein interaction networks with more relationships than other proteins. They also directly interacted with non-HSPs, such as hypoxia up-regulated protein 1, T-complex protein 1 subunit epsilon and stress-induced-phosphoprotein 1. These non-HSPs also interacted with other proteins, such as histone deacetylase 1 and complement component C7. Interaction networks of the differentially expressed proteins from the mammary tissue and liver are shown in Fig 4A and 4B, respectively.

## Discussion

In this study, changes in the abundance of proteins in mammary and hepatic tissue resulting from heat stress were investigated using the iTRAQ proteomic approach. Over 2,700 proteins were identified in each tissue, 80 and 200 proteins were differentially expressed in the mammary tissue and liver, respectively, in response to the heat stress. Most notably there was a dramatic increase in HSPs in the liver in response to heat stress and then were considered as central “hubs” in the interaction networks that were independent of the feed intake reduction seen in heat-stressed cows.

Several previous studies have indicated that milk yield and milk protein content were decreased in heat-stressed compared to control cows [6,8]. In our study, the experimental design ensured the DMI was similar between heat-stressed and pair-fed cows, and milk yield was also decreased in heat-stressed cows [5]. Regarding the milk components, Bernabucci et al. (2010) found that contents of α- and β-casein in bovine milk were decreased, and κ-casein was not different in summer milk when compared with spring milk [31]. Later, they found that casein fractions have the lowest values in the summer and the greatest values in the winter, while the milk whey was greater in summer than in the winter and spring [2]. More recently, decrease in casein concentration and α_s2_-casein number, while increase in milk whey was observed in milk from heat-stressed cows compared with pair-fed control cows [6]. Similarly, in mammary epithelial cells exposed to heat stress in vitro, caseins mRNA was decreased compared to controls [11,32–33]. In our study, casein fractions (αS1-, αS2-, β- and κ-casein) and major milk whey in mammary tissue were not significantly different between heat-stressed and pair-fed cows that were partly similar to the results of the above-mentioned studies in vivo. Although individual milk proteins in mammary gland were not statistically different, the total milk protein content was affected by heat-stress in our study. This phenomenon may indicate lower biosynthesis of milk protein in mammary gland from heat-stressed cows. Several proteins, such as chitinase-3-like protein 1 (CHI3L1) and lysosomal protective protein, were increased in mammary tissue from heat-stressed cows compared with pair-fed control cows in this study. CHI3L1 is a minor milk protein in mammary secretions that mediates mammary tissue remodeling and differentiation [34–35]. More recently, Wall et al. (2013) found expression level of CHI3L1 mRNA was significantly lower in four-times daily milking than two-times daily milking during unilateral frequent milking (first 21 day of lactation), that corresponded to up- and down-regulation of milk yield in paired udder halves, respectively. CHI3L1 mRNA level negatively correlated with milk yield and related to the response of the mammary tissue to the changes in unilateral frequent milking were implicated [36]. In other studies, CHI3L1 has been reported to regulate oxidant injury, antibacterial response and inflammasome activation that was considered as a strong inducer of several signaling pathways including mitogen-activated protein kinase and phosphotidylinositol-3-kinase pathways [37–38]. Thus, we suggested that the increase in CHI3L1 may negatively associated with lactogenesis in the mammary gland and resulted in decrease in milk yield from heat-stressed cows. As a result, we found that significantly decreased milk yieldin heat-stressed cows, compared to pair-fed cows. As discussed previously, we suggested that several proteins increased in the mammary tissue of heat-stressed cows may contribute to increase the immune function and protecting against mammary cells damage. Unfortunately, we could not detect the changes in milk proteome response to heat stress as a complementary, and if necessary, further studies should be performed.

Cows exposed to heat stress have negative energy balance [7,39] and several researchers indicated that loss of body weight and plane of postabsorptive nutrition in heat-stressed cows were related to the negative energy balance [39–40]. To respond to heat stress, cows have a coordinated change in energy supply and utilization by multiple tissues. More recently, differences in metabolites in the serum between spring and hot summer cows were identified by integrated metabolomic and lipidomic approaches that were involved in energy and amino acids metabolic pathways [41]. Regarding the changes in mammary proteome, decreased cytosolic and mitochondrial malate dehydrogenase (MDH1 and MDH2) were identified in heat-stressed cows. They function as key enzymes in the tricarboxylic acid cycle (TCA) for energy metabolism through aerobic respiration. Interestingly, the TCA cycle is considered as a hub for the biosynthesis of lipids, proteins and nucleic acids, as their precursors originate from TCA cycle intermediates [42]. As a result, biosynthesis of nucleic acids and protein was inhibited by heat treatment [40]. However, decreas of MDH1 and MDH2 in mammary tissue of heat-stressed cows was affected by cellular energy state and intermediates in TCA cycle, further investigation is recommended to check subsequent regulation of milk biosynthesis. Regarding the changes in the liver proteome, we found several proteins, such as cytochrome b-c1 complex subunit 6, cytochrome c oxidase subunit 6C, NADH dehydrogenase [ubiquinone] 1 beta subcomplex subunit 6, NADH dehydrogenase [ubiquinone] iron-sulfur protein 8, and cytochrome P450 1A1 and 2A1 were decreased in heat-stressed compared to control cows. Our results were similar to the results of liver proteome of cows exposed to heat stress or cooling conditions during the dry period reported by a previous study [43]. These proteins were assigned into the oxidative phosphorylation pathway and it is well known that they are the components of the electron transport complex [44]. Of these, cytochrome b-c1 complex subunit 6 is an essential component for cytochrome c1 and cytochrome c complex, and reduced cytochrome c oxidase complex activity reduces ATP production and supplying glycolysis for ATP synthesis [45–46]. When ATP production reduced from electron transport complex are not enough to meet the energy requirement of dairy cows, mobilizing adipose and skeletal muscle tissues would be marked by increasing the circulation of cortisol and plasma urea nitrogen levels [47–48]. NADH dehydrogenase [ubiquinone] 1 beta subcomplex subunit 6, one of core subunits of complex I, is related to the stability and activity of complex I. Defect of complex I was linked to oxidative stress [49]. Thus, we put forward a novel hypothesis that decrease in these proteins may contribute to the reduction of energy production and implicating oxidative stress in liver in heat-stressed cows compared to control cows. However, the relationship between decrease in electron transport complex proteins and negative energy balance in the heat-stressed cows needs further investigation.

Furthermore, numerous studies have demonstrated that gene expression of HSPs and secreted proteins are increased in the conditions of heat stress [20–21,50–51]. The HSPs family of proteins is associated with preventing protein denaturation and repairing unstable proteins that are produced by heat stress [52–53]. Thus, HSPs play a cytoprotective role and interact with a variety of cellular proteins [53–54]. Several previous studies found that mRNA levels of HSP70 were increased in the initial period and then gradually decreased in mammary epithelial cells exposed to acute heat stress in vitro [11,32–33]. In addition, increases in the expression of several HSPs were observed in milk cells of dairy goats exposed to heat stress [55]. Unexpectedly, we found that HSPs family was not significantly different in mammary tissue of the cows exposed to heat stress comparative with control cows lasting 9 days. Interestingly, differences in expression levels of HSPs were presented among specific tissue under the heat stress, the gut and liver had been previously reported to be the tissues most sensitive to heat stress [56]. In our study, several HSPs were increased in the liver of heat-stressed cows and were shown to be central “hubs” in the protein interaction networks. Expression of HSPs is linked to the kinetics of thermotolerance acquisition, maintenance, and decay [57]. Thus, the increased expression HSPs may involve in metabolic adaptation to thermal stress and capable of acquired thermotolerance in the liver other than mammary tissue of heat-stressed cows. In addition, a previous study has indicated that HSP accumulation in lipopolysaccharide-stimulated human macrophages attenuated the expression of TNF-alpha mRNA and protein that served as an inflammatory cytokine [58], and as such HSPs may serve as modulating signals for immune and inflammatory responses [59]. As well, HSPs may facilitate antigen presentation in several cells [60]. In accordance, we have also found that several differentially abundant proteins in liver including HSP 90-beta and HSP 90-alpha were involved in the antigen processing and presentation by pathway analysis. As implicated in previous study, we therefore believe that changes in liver proteins may protect against the negative aspects including inflammatory-like condition of cows induced by heat stress.

## Conclusions

In summary, iTRAQ proteomic approach was applied to investigate the liver and mammary proteome profiles from heat-stressed and pair-fed thermal neutral cows. Our results shown that most of the differentially expressed proteins from mammary and liver were all associated with protein metabolic process. Regarding the mammary gland, differentially expressed proteins were related to pyruvate, glyoxylate and dicarboxylate metabolism pathways. Regarding the liver, differentially expressed proteins were associated with oxidative phosphorylation and antigen processing and presentation pathways. In addition, several heat shock proteins had more interaction than other proteins in the interaction network. The findings revealed the changes in mammary and liver proteome of heat-stressed cows independent of feed intake that may help to understand metabolic adaptation to thermal stress and further for exploring the effects of heat stress on milk production and components.

## Supporting information

S1 Fig Western Blot analysis of HSP 90-alpha/beta in liver (A) and mammary tissue (B) of the experimental cows.
(DOCX)

S1 Table Ingredients and chemical composition of diets. (DOCX)

S2 Table The differential expressed proteins in mammary tissue between heat-stressed and pair-fed cows by iTRAQ approach. (XLSX)

S3 Table The differential expressed proteins in liver between heat-stressed and pair-fed cows by iTRAQ approach. (XLSX)
