# Supplementary material for: Heat stress induces proteomic changes in the liver and mammary tissue of dairy cows independent of feed intake: An iTRAQ study
Source: PLoS One. 2019 Jan 9;14(1):e0209182. doi: 10.1371/journal.pone.0209182 (PMC6326702; doi:10.1371/journal.pone.0209182)
Supplement: S1 Fig — Western Blot analysis of HSP 90-alpha/beta in liver (A) and mammary tissue (B) of the experimental cows. (DOCX) [file pone.0209182.s001.docx]

**S1 Fig.** Western Blot analysis of HSP 90-alpha/beta in liver (A) and mammary tissue (B) of the experimental cows

PF

HS


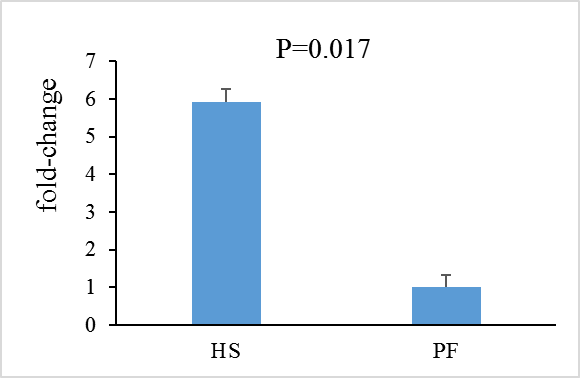

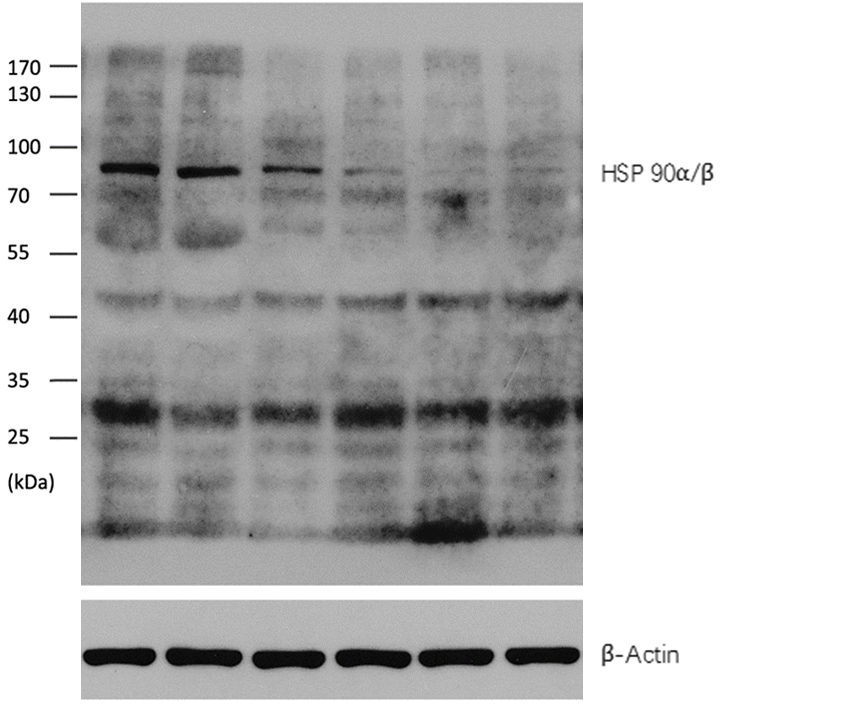


**A**

HS

PF


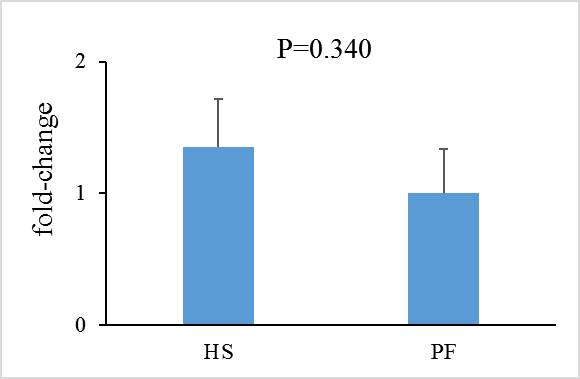

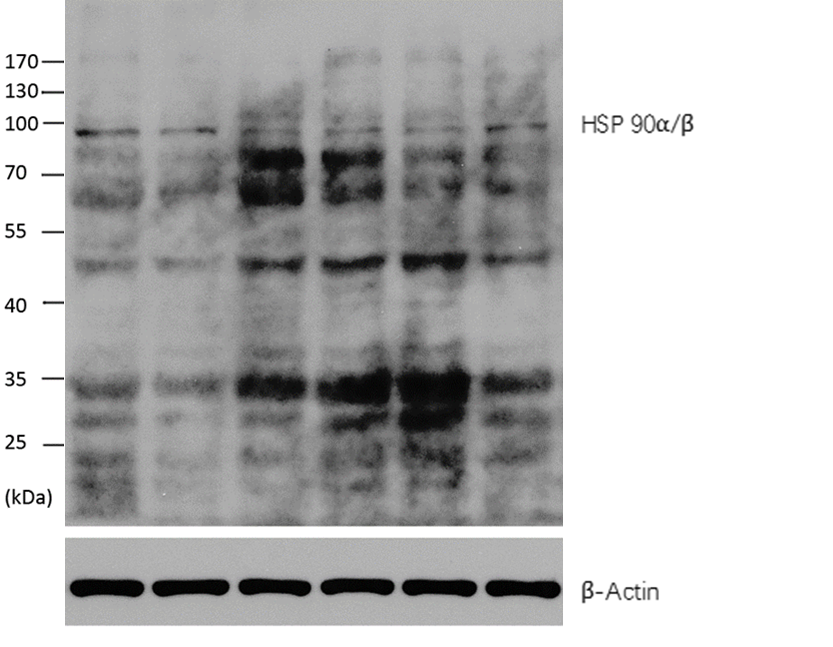


**B**
