# Supplementary material for: Heat stress induces proteomic changes in the liver and mammary tissue of dairy cows independent of feed intake: An iTRAQ study
Source: PLoS One. 2019 Jan 9;14(1):e0209182. doi: 10.1371/journal.pone.0209182 (PMC6326702; doi:10.1371/journal.pone.0209182)
Supplement: S1 Table — (DOCX) [file pone.0209182.s002.docx]

**Supplemental Table 1**. Ingredients and chemical composition of diets^1^

| ^Item^ | ^% of DM^ | ^Item^ | ^% of DM^ | ^SD^ |
| --- | --- | --- | --- | --- |
| ^Ingredient^ |  | ^Chemical analysis^ |  |  |
| ^Alfalfa hay^ | ^23.98^ | ^ADF^ | ^23.28^ | ^0.020^ |
| ^Extruded-soybean^ | ^2.08^ | ^NDF^ | ^43.34^ | ^0.076^ |
| ^Whole corn silage^ | ^26.02^ | ^CP^ | ^15.64^ | ^0.140^ |
| ^Steam-flaked corn^ | ^21.99^ | ^OM^ | ^93.21^ | ^0.007^ |
| ^Bean pulp^ | ^11.38^ | ^DM^ | ^96.97^ | ^0.006^ |
| ^Rapeseed meal^ | ^4.22^ | ^EE^ | ^2.33^ | ^0.004^ |
| ^Feeding corn meal^ | ^8.19^ | ^NE^_L_^, Mcal/kg of DM^ | ^1.69^ |  |
| ^Limestone^ | ^1.19^ |  |  |  |
| ^Salt^ | ^0.38^ |  |  |  |
| ^Supplement 2^ | ^0.57^ |  |  |  |

^1^Values represent an average of samples collected and composited throughout the trial. Diet DM averaged 54.2%.

^2^ Contained (per kilogram of DM) a minimum 250,000 IU of vitamin A; 65,000 IU of vitamin D; 2,100 IU of vitamin E; Fe 400 mg; Cu 540 mg; Zn 2,100 mg; Mn 560 mg; Se 15 mg; I 35 mg; and Co 68 mg.
